# Association between plasma omentin-1 levels in type 2 diabetic patients and peripheral artery disease

**DOI:** 10.1186/s12933-019-0880-7

**Published:** 2019-06-05

**Authors:** Federico Biscetti, Elisabetta Nardella, Nicola Bonadia, Flavia Angelini, Dario Pitocco, Angelo Santoliquido, Marco Filipponi, Raffaele Landolfi, Andrea Flex

**Affiliations:** 1 Fondazione Policlinico Universitario A. Gemelli IRCCS, Largo F. Vito 1, 00168 Roma, Italy; 2 Clinica Medica e Malattie Vascolari, Roma, Italy; 3 Laboratorio di Biologia e Genetica Vascolare, Università Cattolica del Sacro Cuore, Roma, Italy; 4 Medicina d’Urgenza e Pronto Soccorso, Roma, Italy; 5 Diabetologia, Roma, Italy; 6 Università Cattolica del Sacro Cuore, Roma, Italy; 7 Angiologia Columbus, Roma, Italia; 8 Ospedale San Giovanni Battista ACISMOM, Roma, Italia

**Keywords:** Omentin-1, Peripheral artery disease (PAD), Type 2 diabetes, Atherosclerosis

## Abstract

**Background:**

Type-2 diabetes mellitus is one of the major risk factors of atherosclerosis, particularly in peripheral artery disease (PAD). Several studies have documented a correlation between omentin-1 serum levels, atherosclerosis, and cardiovascular diseases. However, a clear link between circulating omentin-1 and PAD in diabetic patients has yet to be established. The aim of this study was to investigate the potential role of omentin-1 in PAD in type-2 diabetic patients.

**Methods:**

In this cross-sectional study, we analyzed omentin-1 serum levels by ELISA in 600 type-2 diabetic patients with (n = 300) and without (n = 300) PAD at Fontaine’s stage II, III, or IV.

**Results:**

We found that omentin-1 serum levels were significantly lower in diabetic patients with PAD than in diabetic controls (29.46 vs 49.24 ng/mL, P < 0.001) and that the levels gradually decreased in proportion to disease severity (P < 0.05). The association between omentin-1 levels and PAD remained significant after adjusting for major risk factors in a multivariate analysis.

**Conclusions:**

Our results suggest that omentin-1 is reduced in type 2 diabetic patients with PAD and that omentin-1 levels are related to disease severity.

## Background

Peripheral artery disease (PAD) represents a frequent manifestation of atherosclerosis disease. Approximately 202 million people worldwide are suffering from lower extremity artery disease [1]. The prevalence of PAD increases with the prevalence of type-2 diabetes mellitus (T2DM), one of the major risk factors of atherosclerosis. Furthermore, PAD has special features and poorer prognosis in diabetic patients than in non-diabetic patients. Patients with T2DM are a high-risk group for developing extensive vascular disease, which adversely affects quality of life. PAD, in these patients, represents an important public health problem, with a significant impact on healthcare and a heavy economic burden [2]. Consequently, early diagnosis and management of PAD in T2DM patients are critical for reducing the risk of major adverse cardiovascular events (MACE) and major adverse limb events (MALE), minimizing the risk of long-term disability [3]. International guidelines [4] addressing diagnosis, treatment, and overall management of patients with PAD suggest the ankle–brachial index (ABI) as an initial diagnostic test for PAD. However, ABI evaluation has a low sensitivity for detecting the initial stages of PAD, and it may not be applicable in patients with diabetes because of calcification of the artery walls, which can increase vessel stiffness [5].

Therefore, to be able to make an early diagnosis, we need to identify novel biomarkers that can detect PAD among diabetic patients.

In the past few years, attention has been focused on adipokines, cytokines produced and secreted by visceral adipose tissue and involved in the development of atherosclerotic disease. Studies have shown that some of these cytokines take part in the regulation of adipose tissue with a pro- or anti-inflammatory effect, and several researchers are investigating their possible role as biomarkers for metabolic disorders [6]. In this context, findings have documented a possible role of omentin serum levels in cardiovascular diseases. Omentin, a type of adipokine, is a hydrophilic protein with a molecular weight of 40 kDa; it is composed of 313 amino acids and is encoded by a gene present in chromosomal region 1q22–q23, which is related to T2DM [7]. This protein’s mRNA is expressed mainly in the fraction of the vascular stroma of the visceral adipose tissue, and poorly in subcutaneous adipose tissue and mature adipocytes. Omentin mRNA has also been identified in other tissues, such as endothelial cells, epicardial adipose tissue [8], thymus, small intestine, colon, reticulocytes, ovary, lung, and placenta. There are two main homologous isoforms: omentin-1, the most common form in human plasma; and omentin-2, which shares 83% of amino acids with isoform 1 [9]. Currently, the omentin-binding receptors are unknown [10]. Basic and clinical research has documented an anti-inflammatory action of omentin-1 and a mainly negative correlation between its plasma levels and insulin resistance, diabetes, obesity, and metabolic syndrome. Yoo and coworkers and Liu and colleagues, in cross-sectional studies, have documented that omentin-1 levels are reduced in patients with T2DM and metabolic syndrome, especially in patients with carotid plaques, compared to healthy subjects, diabetic subjects, or patients with metabolic syndrome without carotid atheromasia [11, 12]. More recent data have shown that, in general, the reduction of plasma levels of omentin increases the risk of diabetes and of atherosclerotic complications [13]. These observations have suggested a possible role of omentin-1 in atherosclerotic disease.

Other studies have investigated the relationship between omentin-1 and coronary artery disease [14, 15], stroke, and complications of T2DM [13, 16]. Based on these findings, some controversial data emerged about a negative, positive, or independent correlation between omentin-1 levels and cardiovascular risk factors. The analysis of these results revealed a different behavior of omentin-1 in healthy subjects compared to subjects with pre-existing metabolic disease [17, 18].

Only few, non-conclusive data exist regarding omentin-1 and PAD in non-diabetic patients [19] and a definitive link between circulating omentin-1 and PAD in diabetic patients has not yet been established.

The aim of this study was to investigate the potential role of omentin-1 in PAD of type-2 diabetic patients.

## Methods

### Study population

We performed a cross-sectional study of diabetic patients consecutively admitted to the Department of Vascular Diseases of the Fondazione Policlinico Universitario A. Gemelli IRCCS, Roma, Italy, from 1 October 2015 to 31 June 2018. To be enrolled in the present study, each patient had to fulfill the inclusion criteria shown in Table 1. We enrolled diabetic patients with or without PAD. Type-2 diabetes mellitus was defined as a fasting plasma glucose level ≥ 126 mg/dl and/or a HbA1c level ≥ 6.5% or as a medical history of diabetes plus treatment with diabetes medication. Each patient enrolled in the study was assessed by a history of PAD symptoms or a confirmed PAD diagnosis, according to previous criteria established by the Ad Hoc Committee on Reporting Standards of the Society for Vascular Surgery and the International Society for Cardiovascular Surgery [20, 21]. Patients with clinical findings consistent with PAD underwent ABI measurement, and both lower limbs were assessed using arterial Doppler-enhanced ultrasonography, computed tomography angiography, lower limb angiography, at the attending physician’s judgment. Patients with an ABI > 0.90 and without symptoms of PAD did not undergo further testing and were deemed to be without PAD.

**Table 1:** Inclusion and exclusion criteria

Inclusion criteria	Exclusion criteria
Age older than 40 affected by type 2 diabetes mellitus	Inability or refusal to sign informed consent for inclusion of the study
	Renal failure with eGFR < 30 ml/min
	State of pregnancy established or presumed
	History of solid or hematological neoplasia or active neoplasia
	Transplant holder of a solid organ or subjected to bone marrow transplantation
	Gastro-intestinal resection
	Unfavorable prognosis at the judgment of the clinician, or life expectancy of less than 12 months
	Autoimmune or chronic inflammatory pathology
	Confirmed liver cirrhosis with Child–Pugh C functional impairment
	Confirmed or suspected monogenic hereditary dyslipidemia
	Confirmed acquired platelets or congenital platelets disease
	Confirmed congenital hemorrhagic diathesis or acquired coagulopathies; Congenital or acquired thrombophilia

The extent of PAD was determined by using the Fontaine classification, which defines four stages: stage I, asymptomatic; stage II, intermittent claudication; stage III, rest pain; stage IV, ischemic ulcers or gangrene [22].

Patients enrolled were also assessed by a history of heart failure symptoms or a confirmed diagnosis, as previously described [23].

The study was approved by the Ethics Committee of the Fondazione Policlinico Universitario A. Gemelli IRCCS and adhered to the principles of the Declaration of Helsinki. All patients enrolled gave their informed consent.

### Biochemical measurements

All patients enrolled underwent a blood test after an overnight fasting period of 8 h. For every patient, fasting glucose, triglycerides, total cholesterol, and low- and high-density lipoprotein were determined. Renal function was assessed using estimated glomerular filtration rate (eGFR), which was calculated using the modification of diet in renal disease (MDRD) formula. Serum obtained and separated by centrifugation of blood samples was stored at − 80 °C before every measurement. Serum omentin-1 levels were determined by a commercially available ELISA kit (E-EL-H2028, Elabscience) according to its protocol. The intra- and inter-assay coefficients of variation were 3.5% and 10.5%, respectively. The sensitivity, defined as the mean ± 3 SD of the 0 standard, was calculated to be 0.15 pmol/ml. For each patient, the serum levels were measured twice, and the results were averaged.

### Statistical analysis

Demographic and clinical data of the groups were compared using a Chi squared test and a t-test. Omentin-1 serum levels were compared with a Mann–Whitney, Kruskal–Wallis and Dunn’s Multiple Comparison, when appropriate. A log transformation was applied to the not normally distributed variables (fasting glucose, glycated hemoglobin, triglyceride, and omentin-1 levels) prior to performing further analysis. A multivariate stepwise logistic regression analysis was performed, adjusted for traditional risk factors and omentin-1 levels. The area under the receiver operating characteristics (ROC) curve was calculated to test the predictive discrimination of PAD. All analyses were performed using STATA version 11.0 for Windows (Statistics/Data Analysis, Stata Corporation, College Station, TX, USA). Statistical significance was established at P < 0.05.

## Results

The demographic and clinical characteristics of diabetic patients with PAD (indicated as PAD in Table 2) and without PAD (WPAD) are summarized in Table 2. Among the 600 diabetic patients enrolled in the study, 300 were included as PAD, and 300 as WPAD. PAD patients were more often smokers (P = 0.011), had higher blood pressure values (P = 0.010), had more frequent coronary artery disease (defined as a history of ischemic heart disease and/or previous coronary revascularization) (P = 0.022), and had higher LDL-cholesterol values (P = 0.02) than WPAD patients. There were no significant differences between groups regarding sex (P = 0.66), age (P = 0.19), body mass index (BMI) (P = 0.83), heart failure (P = 0.82), mean duration of diabetes (P = 0.48), fasting glucose (P = 0.76), glycated hemoglobin (P = 0.76), eGFR (P = 0.32), total cholesterol (TC) (P = 0.78), HDL-cholesterol (P = 0.59), and triglyceride (P = 0.78). No statistical difference in terms of diabetic therapy was observed between the two patient groups. According to the Fontaine’s classification, 168 patients were defined as stage II, 72 as stage III, and 60 as stage IV.

**Table 2:** Demographic and clinical data of diabetic subjects with and without PAD

	WPAD (n = 300)	PAD (n = 300)	P value
ABI (years ± SD)	1.12 ± 0.5	0.67 ± 0.3	0.009
Men/female (n)	199:101	210:90	0.66
Age (years ± SD)	73.2 ± 9.1	76.1 ± 5.1	0.19
BMI (kg/m^2^)	26.2 ± 3.1	25.6 ± 4.2	0.83
Smoking (current) (%)	84 (28.0)	156 (52.0)	0.011
Hypertension (%)	158 (52.6)	201 (67.0)	0.010
CAD (%)	101 (33.6)	186 (62.0)	0.022
Heart failure (%)	33 (11.0)	38 (12.7)	0.82
Diabetes duration (years ± SD)	10.1 ± 2.1	11.3 ± 4.1	0.48
Total cholesterol (mmol/l)	5.54 (1.12)	5.98 (1.28)	0.78
HDL-C (mmol/l)	1.41 (1.13)	1.31 (1.14)	0.59
LDL-C (mmol/l)	2.13 (1.22)	2.97 (1.23)	0.02
Triglyceride (mmol/l)	2.12 (1.42)	2.33 (1.47)	0.78
Fasting glucose (mmol/l)	7.22 (1.13)	7.22 (1.43)	0.76
Glycated hemoglobin (%)	7.18 (1.87)	7.94 (1.87)	0.76
eGFR (ml/min per 1.73 m^2^)	69.23 (11.03)	65.92 (9.12)	0.32
Treatment			
Diet only (%)	44 (14.6)	31 (10.3)	0.36
Oral agents (%)	164 (54.6)	175 (58.3)	0.87
Insulin therapy (%)	92 (30.6)	125 (41.6)	0.12
PAD			
1-Fontaine’s II (%)		168 (56.0)	
2-Fontaine’s III (%)		72 (24.0	
2-Fontaine’s IV (%)		60 (20.0)	

**Fig. 1:**
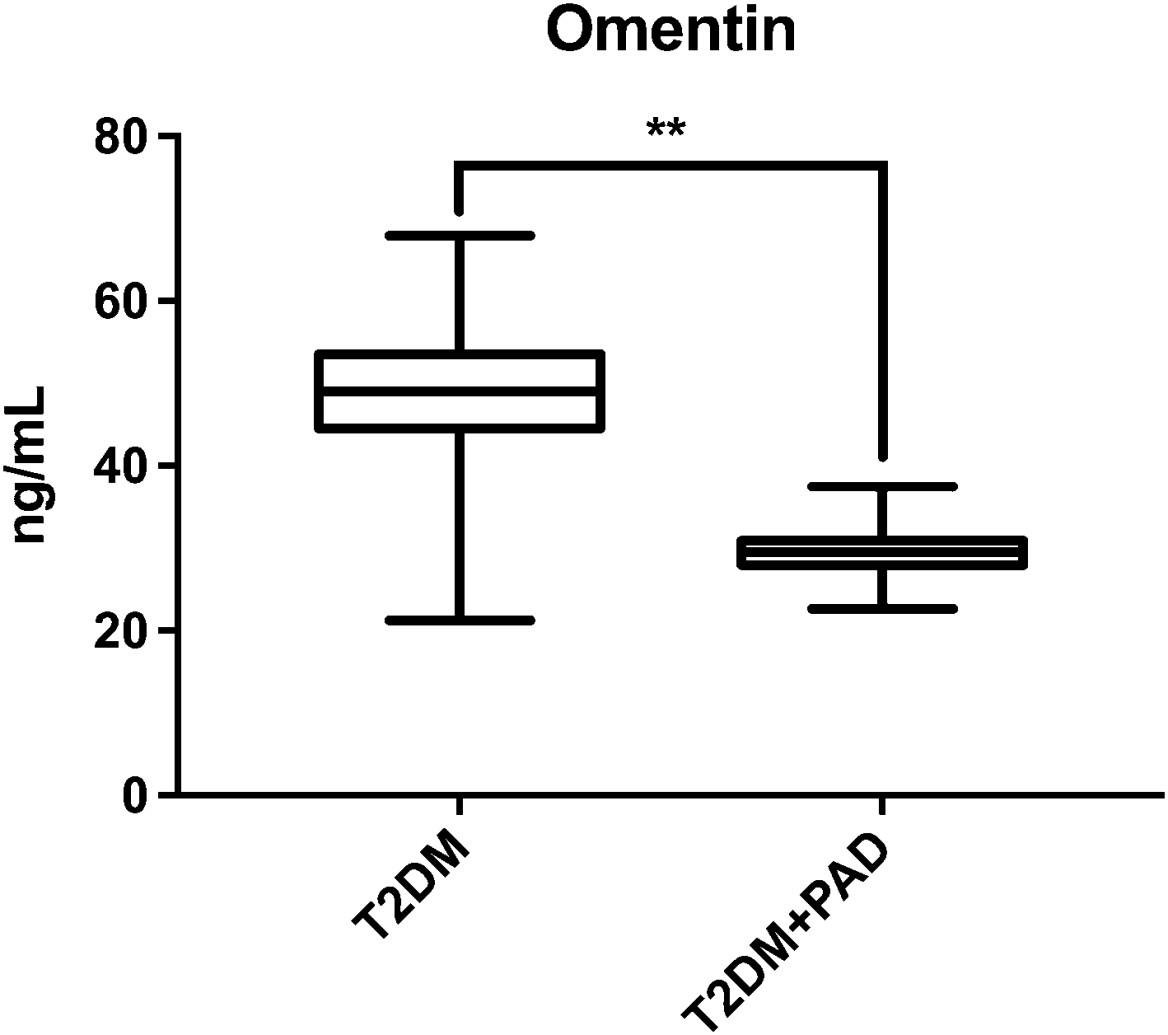
Omentin-1 levels according to PAD diagnosis. On the box plots, central lines represent the median, the length of the box represents the interquartile range and the lines extend to minimum and maximum values. Omentin-1 levels were lower among patients with PAD than among those without PAD (**P < 0.001)

**Fig. 2:**
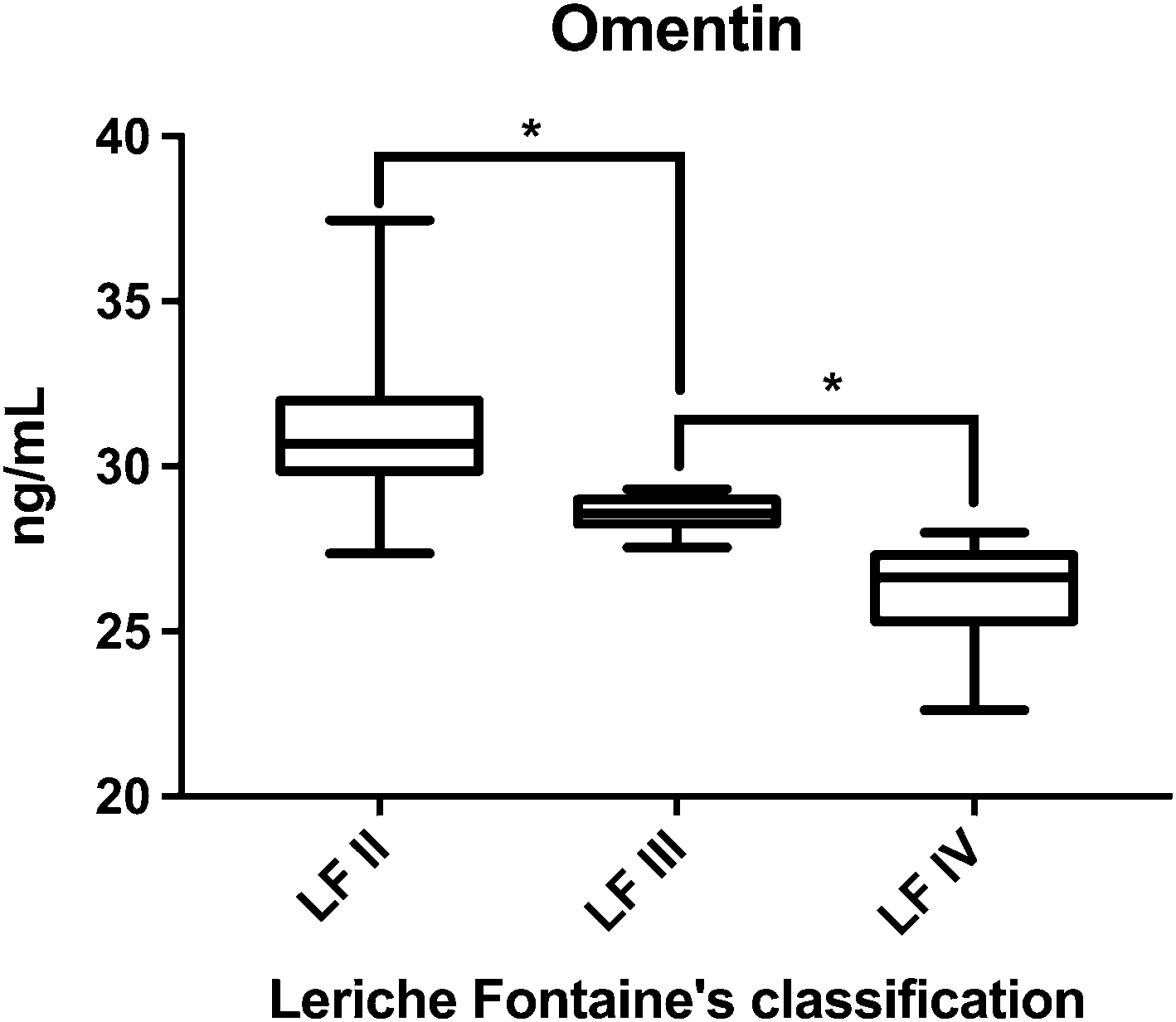
Omentin-1 levels according to PAD severity. On the box plots, central lines represent the median, the length of the box represents the interquartile range and the lines extend to minimum and maximum values. According to patients’ functional status, represented by the Leriche-Fontaine classification, lower levels of circulating omentin-1 in patients with more severe disease were detected (LF II vs LF III, *P < 0.05; LF III *vs* LF IV, *P < 0.05)

Omentin-1 levels were lower among patients with PAD (29.46 ± 2.32 ng/ml) than among those without PAD (49.24 ± 6.44 ng/ml), as highlighted in Fig. 1. Moreover, when evaluating omentin-1 concentration according to patients’ functional status, we observed a distinct trend, with lower levels of circulating omentin-1 in patients with more severe disease (Fig. 2).

The multivariate logistic regression analysis showed that, after adjustments for the cardiovascular risk factors age, male sex, BMI, eGFR, smoking, hypertension, ABI, CAD, heart failure, LDL-cholesterol, and omentin-1 levels, only ABI (OR 7.12, P = 0.011) and LDL-cholesterol (OR 2.37, P = 0.025) were independent determinants for the presence of PAD in patients with T2DM. Interestingly, there was an inverse relationship between omentin-1 levels and PAD in our diabetic population (OR 0.90, P < 0.001) (Table 3).

**Table 3:** Multivariable stepwise logistic regression model for presence of PAD adjusted for common risk factors and for omentin-1

	Variable OR (95% CI)	z	P value
ABI	7.12 (2.44–9.51)	3.31	0.011
LDL-cholesterol	3.12 (1.18–4.28)	2.43	0.015
Omentin	0.88 (0.77–0.96)	− 5.11	< 0.001

**Fig. 3:**
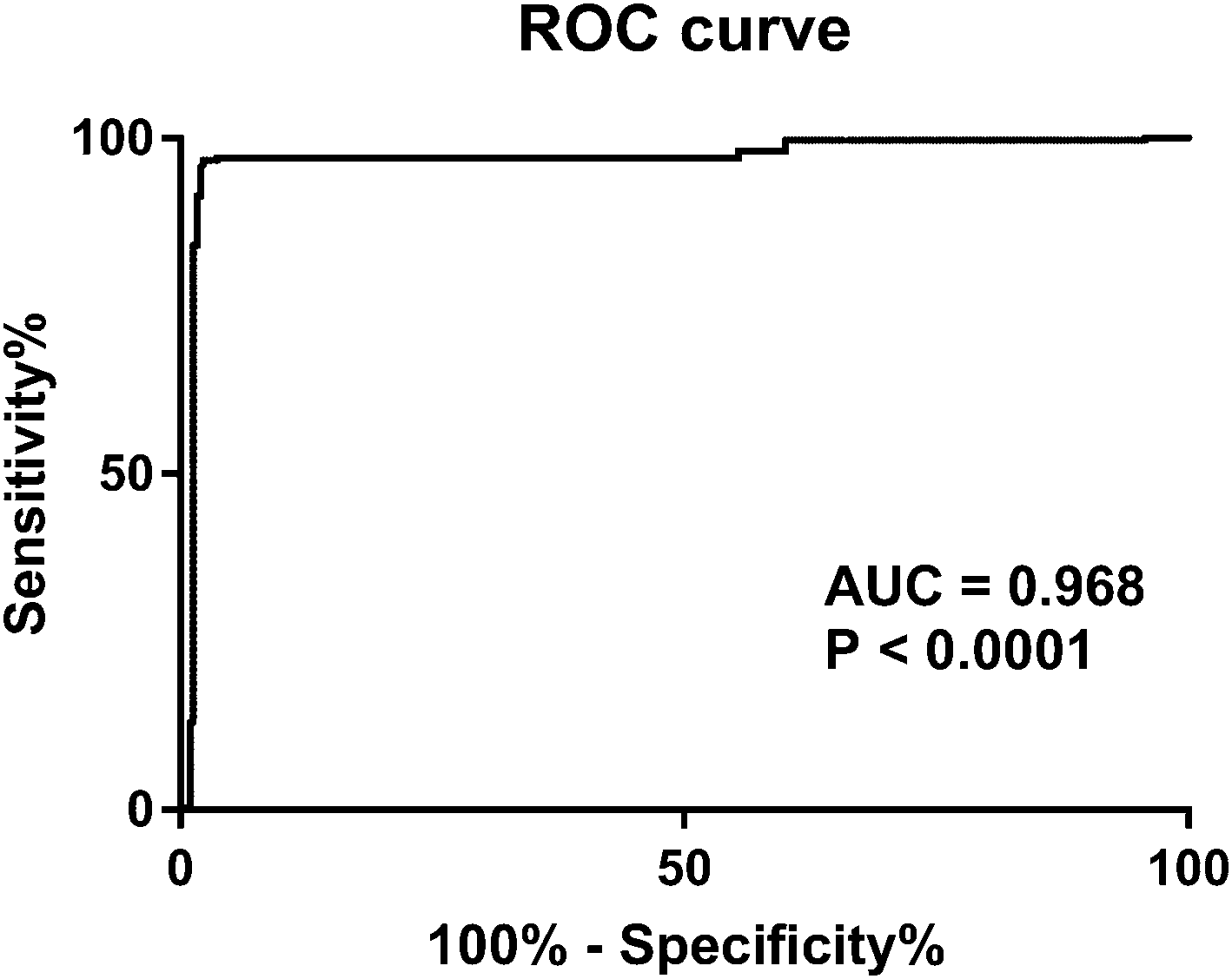
ROC curve analysis of the ability of omentin-1 to predict the presence of PAD in diabetic patients. The ability of the area under the ROC curve was 0.968

The ability of the area under the ROC curve based on omentin-1 levels to predict the presence of PAD in diabetic patients was 0.968 (Fig. 3) and the best cut-off value of omentin-1 for prediction of the occurrence of PAD in our population was < 37.57 ng/ml (Sensitivity 97%, Specificity 96.33%).

## Discussion

Peripheral arterial disease is an endemic problem, with high direct and indirect social costs. Patients with PAD are often unable to work, require frequent hospitalizations, and often undergo major amputations [20, 24–26]. Considering the relevance of the disease, the ability to make an early diagnosis of PAD in diabetic patients is becoming increasingly important. Of the various diagnostic tests available, none of them is currently sensitive and specific enough to make an early diagnosis, and often the patient consults the specialist when the disease is already well advanced.

Inflammation plays a fundamental role in the formation of atherosclerotic plaque, particularly in the diabetic patient. A state of latent chronic inflammation can result from the initiation and progression of atherosclerotic disease. This inflammation is linked to different pathways, is mediated by hyperglycemia, and altered by the oxidation–reduction status and the release of inflammatory cytokines [27–29]. Adipose tissue is a very important source of inflammation, through the secretion of cytokines that directly or indirectly promote inflammatory pathways [30]. Among the various cytokines produced by adipose tissue, adipokines represent a pool responsible for various detrimental or protective processes [6, 30, 31]. For this reason, we wanted to evaluate the role of an easy-to-measure adipokine in diabetic patients. We found that omentin-1 is reduced in diabetic patients with PAD and that omentin-1 serum levels are statistically significantly lower in T2DM patients with PAD than in diabetic patients without PAD. This result is very interesting because the relationship between omentin and PAD in T2DM has never been documented before. In fact, Onur and colleagues have evaluated, in a cross-sectional and observational study, the association between the levels of omentin-1 and PAD of the lower limbs, demonstrating how the serum levels of this adipokine are lower in patients affected by PAD compared to healthy controls [19]. This study confirms that this adipokine is not only part of the energy balance but has a protective effect in diabetic patients, being involved in the lipid metabolism and inflammation that cause the vascular complications of diabetes. As already mentioned, omentin-1 levels are negatively associated with diabetes and metabolic syndrome and are reduced in diabetic patients with carotid atherosclerosis [11, 12]. The protective effect of omentin-1 could be explained by the suppression of inflammation and apoptosis of endothelial cells [6, 32]. To our knowledge, this is the first time that reduced omentin-1 serum levels were assessed as a potential biomarker for PAD in a diabetic population. Furthermore, we also demonstrated that omentin-1 serum levels decrease according to disease severity. This is a further important finding because, within the diabetic population affected by PAD, the values of omentin-1 could help to stratify patients to facilitate a more appropriate diagnostic and therapeutic process. Finally, we have documented that the relationship between lower omentin-1 levels and PAD in T2DM remains significant also after adjustment for potential confounding variables such as age, smoking status, hypertension, CAD, heart failure and serum lipid profile. If such a result is confirmed, the determination of omentin-1 serum levels could prove to be a new biomarker for early diagnosis and an effective follow-up of PAD in diabetic patients.

A limitation of our study is that its cross-sectional nature is not able to establish causal relationships between the findings. We need prospective data to confirm these results and to examine whether lower omentin-1 levels may also suffice as an effective biomarker for PAD in patients with type-2 diabetes. A further limitation is that we did not use a healthy control population to determine normal levels of omentin-1. In fact, there is no unequivocal evidence regarding the normal levels of omentin-1 [33–35]. Another limitation of the study is that we have not considered the distribution of adipose tissue in patients, and it is conceivable that a different distribution of fat, other than the one measured by the simple body mass index, could influence the levels of omentin-1. A further confounding factor is that we have not considered therapy among the significant variables, and it is possible that statins and hypoglycemic agents can play a role in the homeostasis of this adipokine. An additional limitation of our study is that we have not made a distinction between the type of antidiabetic therapy and the levels of omentin-1. Furthermore, it was not possible to study a relationship between aerobic exercise and the levels of this adipokine. Indeed, Menzel and coworkers suggest how different metabolic conditions can influence omentin levels, and this might also have happened in our model [17]. Finally, genetic analysis could help to better define the relationship between omentin-1 and PAD in diabetic patients. In fact, Jamshidi and colleagues evaluated the correlation between the polymorphism of the omentin Val109Asp gene—a missense variant of exon 4—and the risk of coronary artery disease [36]. Such a study of polymorphisms in our population could potentially provide interesting data.

## Conclusion

In conclusion, we have shown that a relationship exists between omentin-1 levels and the presence of PAD in a diabetic population, that omentin-1 levels are reduced in T2DM affected by PAD, and that omentin-1 levels correlate with disease severity. Although further confirmations are necessary, these findings could foster earlier diagnosis and better management of this widespread disease.

## Data Availability

Not applicable.

## Abbreviations

ABI: ankle–brachial index
BMI: body mass index
CT: computed tomography
CAD: coronary artery disease
eGFR: estimated glomerular filtration rate
HDL: high-density lipoprotein
LDL: low-density lipoprotein
MACE: major adverse cardiovascular events
MALE: major adverse limb events
PAD: peripheral artery disease
ROC: receiver-operating characteristics
T2DM: type 2 diabetes mellitus
VLDL: very low-density lipoprotein
WPAD: without PAD
